# Supplementary material for: Effect of one dose of ceftriaxone during endotracheal intubation on the incidence of aspiration pneumonia in cerebral hemorrhage patients: A randomized, controlled, double-blind clinical study protocol
Source: PLoS One. 2025 Jan 14;20(1):e0316811. doi: 10.1371/journal.pone.0316811 (PMC11731750; doi:10.1371/journal.pone.0316811)
Supplement: Attachment — Submitted filename: recommendations.docx [file pone.0316811.s001.docx]

**Recommendations**

1) There is a mistake in the date in methods and analysis (line 41) :it should be from April 2023 to April 2024.
2) Please provide a copy of ethical committee approval for the trial.
