# Supplementary material for: Effect of one dose of ceftriaxone during endotracheal intubation on the incidence of aspiration pneumonia in cerebral hemorrhage patients: A randomized, controlled, double-blind clinical study protocol
Source: PLoS One. 2025 Jan 14;20(1):e0316811. doi: 10.1371/journal.pone.0316811 (PMC11731750; doi:10.1371/journal.pone.0316811)
Supplement: Attachment — Submitted filename: Response to Reviewers.docx [file pone.0316811.s002.docx]

Dear *PLOS One* Editors,

Thank you for your thoughtful comments and suggestions for our manuscript, “Effect of one dose of ceftriaxone during endotracheal intubation on the incidence of aspiration pneumonia in cerebral hemorrhage patients: A randomized, controlled, double-blind clinical study protocol”. Below in blue are responses to Editor and Reviewer comments that you will also find addressed in our manuscript. We appreciate the opportunity to address the comments and resubmit an improved manuscript for considered publication in *PLOS One*.

Sincerely,

Xuan Song, MD, PhD

**Comments to the Author**

Reviewer #1: I thank the editor for submitting the work of the Chinese group for review.

This work consists of a double-blind randomized controlled trial protocol that seeks to evaluate whether it is possible to decrease the incidence, with a single dose of cephalosporin, of aspiration pneumonia in a specific test population, those subject to intracerebral hemorrhage.

Keywords.

1. I congratulate the authors for their choice of keywords. All relevant and found in the reference database.

Thank you.

Introduction

2. To check reference #1, as wording or citation.

This has been addressed.

3. Line 66-74 I advise the authors, in this part of the introduction to include some reference to the sentences said, or, if they are speculations or statements of the authors perhaps rephrase the sentences.

We have added references to these sentences.

4. Line 76-85 I advise the authors in this passage to specify the type of patients examined by the studies mentioned, for the study in reference no. 2 ALL emergency intubated patients were taken, for studies instead 3 and 4 patients were examined, for study 3 elderly, after the age of 70 years stated, and for study 4 outcome of a population with a median age of 78 years is evaluated.

This is a point to be specified in the introduction for clarity and accuracy.

We have specified the types of patients in the above studies in the introduction.

4. Line 116-121 To be included references to this. They are not in the document.

We have added references to these sentences.

Study design

6. I would like to ask the authors how they plan to deal with COPD patients, patients who are more delicate and who may also be more prone to pneumonia, both nosocomial but especially community-acquired pneumonia. Will they be excluded or included in the study and then perhaps analyzed separately? This is a subcategory to be carefully evaluated.

These patients are not excluded, but will be compared in baseline characteristics. We have noted this in the manuscript.

Figure 1 and 2

7. Regarding the figures I would have some clarifications for the authors to make. Figure 1 is interesting in that it describes, adequately, the pattern of enrollment, interventions, and assessments done.

Thank you.

8. For Figure 2, it would also be to complete after the intervention and control boxes, with perhaps the patients lost and the amount of patients analyzed.

Thank you for your suggestion. We have revised Figure 2 according to the steps of the randomized controlled trial.

9. Regarding exclusion criterion 7, however, I would specify the reason for this criterion. Why were those who had a history of alcohol consumption in the week prior to endotracheal intubation not considered? For what reason? Is there a reference that discourages this type of patient in the possibility of being enrolled?

Thank you for your question. The use of cephalosporin after drinking alcohol can easily cause disulfiram reaction.The manifestation of CIDLR (cephalosporin-induced disulfiram-like reaction) varies from mild reaction like facial flushing, nausea or vomiting, to a severe reaction including angioedema, hypotension, shock, or death (1,2). Therefore, we excluded such patients in order to avoid risk.

(1) Mergenhagen KA, Wattengel BA, Skelly MK, Clark CM, Russo TA. Fact versus Fiction: a Review of the Evidence behind Alcohol and Antibiotic Interactions. *Antimicrob Agents Chemother*. 2020;64(3):e02167-19. Published 2020 Feb 21. doi:10.1128/AAC.02167-19

(2) Ren S, Cao Y, Zhang X, Jiao S, Qian S, Liu P. Cephalosporin induced disulfiram-like reaction: a retrospective review of 78 cases. *Int Surg*. 2014;99(2):142-146. doi:10.9738/INTSURG-D-13-00086.1

Intervention

10. Line 162-163 Why was the infusion rate of ceftriaxone chosen to be 1 hour and not 30 minutes given that ceftriaxone is known to be a time-dependent drug and acts on the plasma peak and not on maintaining a constant dose? This is a point that needs to be evaluated and clarified in order to be timely in describing the intervention, which is also a cornerstone on which the study is based, the administration of the antibiotic.

Thank you for your question. According to the drug instructions of ceftriaxone sodium, the infusion time should be at least 30min to avoid adverse reactions, so we defined the infusion time as 1 hour in the protocol.

11. Line 168. I advise authors to state serious adverse events and not take them for granted, I assume symptoms of severe allergic crisis in patients not known allergic, but to be stated.

We now include this information in the manuscript.

Data collection

12. I would advise the authors to subdivide this subsection further, perhaps putting, patient characteristics, intubation characteristics, and antibiotic therapy. So that the data collected can be visualized better and more schematically.

Agree with the reviewer. We have subdivided Data Collection into the above sections.

13. Additional data that I would also collect are laboratory tests at admission, at least those related to infectious status, considering that later we are going to evaluate an infection, such as (Body temperature, complete hemogram with complete Leukocyte formula, Thrombus and CRP and procalcitonin, and maybe other values if present and measured), so that we have a start and know possibly whether to exclude from the study patients who do not have obvious fever but maybe had an initial infectious status. And possibly even if there is a possibility, of a bronchial secretion analysis at the time of intubation, or even better a BAL.

Agree with the reviewer. We have included laboratory test results in the information to be collected.

Statistical analysis

14. I recommend that the authors also state that the data will be described statistically with mean and standard deviation or median and quartile depending on the distribution.

Agree with the reviewer. We have included this information in the manuscript.

Discussion

15. Line 276-278 Appropriate reference to the authors' statement should be included.

Reviewer #2: 1) There is a mistake in the date in methods and analysis (line 41) :it should be from April 2023 to April 2024.

Thank you. We have corrected this mistake.

2) Please provide a copy of ethical committee approval for the trial.

Ok, we submitted the copy of ethical committee approval of the trial.

Reviewer #3: Dear Authors, Thank you for giving us the opportunity to review this article titled “Effect of one dose of ceftriaxone during endotracheal intubation on the incidence of aspiration pneumonia in cerebral hemorrhage patients: A randomized, controlled, double-blind clinical study protocol”.

I don't have any conflict of interest.

I have some concern for this manuscript.

First of all, this should be clarified. Should antibiotics be given before or after intubation? In some sentences, antibiotics are mentioned before intubation and in others after.

Due to the urgency of rescuing patients in clinical practice, we cannot guarantee the use of ceftriaxone before tracheal intubation. According to our protocol, it must be used within 2 hours after tracheal intubation.

The introduction part is too long.

We have endeavored to shorten the introduction.

bacteria should be given briefly.

We have endeavored to shorten the bacteria discussion in the introduction, but feel it is important to note the kinds of bacteria that are found in aspiration pneumonia cases.

exclusion criteria

consciousness changes?

Actually, isn't this the riskiest group?

Therefore, informed consent should be obtained from their relatives. I think excluding the most risky patient group will impoverish this study.

I can't agree with you more. We included patients with consciousness changes in our protocol and primarily excluded those who had an impact on aspiration and aspiration pneumonia. We seek informed consent from their relatives with consciousness changes.

History of drinking in the week before endotracheal intubation. (why? reference?)

Thank you for your question. The use of cephalosporin after drinking alcohol can easily cause disulfiram reaction.The manifestation of CIDLR (cephalosporin-induced disulfiram-like reaction) varies from mild reaction like facial flushing, nausea or vomiting, to a severe reaction including angioedema, hypotension, shock, or death (1,2). Therefore, we excluded such patients in order to avoid risk.

(1) Mergenhagen KA, Wattengel BA, Skelly MK, Clark CM, Russo TA. Fact versus Fiction: a Review of the Evidence behind Alcohol and Antibiotic Interactions. *Antimicrob Agents Chemother*. 2020;64(3):e02167-19. Published 2020 Feb 21. doi:10.1128/AAC.02167-19

(2) Ren S, Cao Y, Zhang X, Jiao S, Qian S, Liu P. Cephalosporin induced disulfiram-like reaction: a retrospective review of 78 cases. *Int Surg*. 2014;99(2):142-146. doi:10.9738/INTSURG-D-13-00086.1

Why is ceftriaxone given as an infusion for 1 hour?

Thank you for your question. According to the drug instructions of ceftriaxone sodium, the infusion time should be at least 30min to avoid adverse reactions, so we defined the infusion time as 1 hour in the protocol.

Will each patient be paag after intubation? to exclude old infiltrates?

Thank you for your question. According to the needs of the patient.

data collection

Has a mechanical application been made or not to prevent aspiration?

Will this information be recorded?

Thank you for your question. We studied aspiration pneumonia due to glottis exposure during endotracheal intubation. After endotracheal intubation, both groups will be treated with the usual procedures to prevent aspiration. These measures include: elevation of the head of the bed, good oral care, swallowing function assessment and training, comprehensive rehabilitation, nasogastric feeding, improvement of gastric emptying function, and monitoring of gastric residual volume.

The discussion part should be completely rewritten.

We have endeavored to rewrite the discussion.

And, The most important question that needs to be explained is this.

Why cerebral hemorrhage patient group?

Thank you for your question. There are two main reasons. First, due to the characteristics of the hospital, there are many patients with cerebral hemorrhage. Secondly, patients with cerebral hemorrhage often experience such complications as consciousness disorders, respiratory center damage, airway obstruction. Endotracheal intubation is often required to protect the airway and maintain oxygenation.
