# Supplementary material for: Effect of one dose of ceftriaxone during endotracheal intubation on the incidence of aspiration pneumonia in cerebral hemorrhage patients: A randomized, controlled, double-blind clinical study protocol
Source: PLoS One. 2025 Jan 14;20(1):e0316811. doi: 10.1371/journal.pone.0316811 (PMC11731750; doi:10.1371/journal.pone.0316811)
Supplement: Attachment — Submitted filename: Response Letter.docx [file pone.0316811.s003.docx]

Reviewer #4: I thank the editor for giving me the opportunity to review the revised manuscript titled Effect of one dose of ceftriaxone during endotracheal intubation on the incidence of aspiration pneumonia in cerebral hemorrhage patients: A randomized, controlled, double-blind clinical study protocol by Xuan Song and others from China The concept for this study Protocol is praise worthy and covered all relevant aspects for the study The protocol is meticulously planned with the inputs from the reviewer it is further improved it will lead to meaningful outcome The methodology is replicable well written

I have gone through the Manuscript and Reviewer's comments and I have found the authors have made changes asper the recommendations of the reviewer the authors answered all the quarries put by reviewer . With these changes in the manuscript it is fit for acceptance without any further modification

We extend our sincere gratitude to the reviewer for their positive and constructive review of our manuscript. Your insightful comments and feedback have been invaluable, and we appreciate the time and effort you dedicated to evaluating our work.

Reviewer #5: While I appreciate the authors' efforts to address limitations in their revised manuscript, serious concerns persist. The continued conflation of aspiration pneumonia and aspiration pneumonitis raises validity issues. Misdiagnosis and inappropriate antibiotic use in pneumonitis cases are major concerns, it has worsened the problem of MDR organisms worldwide as outlined by major guidelines. Implementing additional diagnostic measures, such as bronchoscopy, or lab markers like procalcitonin is crucial to distinguish between these entities before deciding on preemptive antibiotic therapy.

To address these concerns and ensure a robust study, I recommend refining the definition of aspiration pneumonia and clearly defining pneumonitis vs pneumonia, and possibly utilizing additional diagnostic measures. Additionally, authors should provide more data supporting the efficacy of single-dose ceftriaxone specifically for aspiration pneumonia prevention in ICH patients. Before potentially exposing patients to unnecessary risk of inappropriate antibiotic use, authors should conduct a cost-effectiveness analysis to assess the balance between potential benefits and risks associated with prophylactic ceftriaxone use.

We would like to express our appreciation to the reviewer for their meticulous examination of our manuscript and the thoughtful feedback provided. Their careful review has been instrumental in shaping and improving our work. Aspiration pneumonitis (Mendelson’s syndrome) is a chemical injury caused by the inhalation of sterile gastric contents, whereas aspiration pneumonia is an infectious process caused by the inhalation of oropharyngeal secretions that are colonized by pathogenic bacteria. Our protocol refers to aspiration pneumonia. We have further clarified the difference between aspiration pneumonia and aspiration pneumonitis in the manuscript. We have also added a paragraph to the discussion about the benefits vs. potential risks of prophylactic antibiotic use (i.e., antibiotic resistance).

Furthermore, the single-center design and small sample size, previously acknowledged by the authors, further limit the generalizability and impact of this research. Given the importance of aspiration pneumonia, it is vital that studies addressing this issue are conducted with the utmost rigor and precision. Finally, collaborating with other centers could enhance the study's generalizability and impact. Unfortunately, the current manuscript falls short of this standard.

We appreciate the thoughtful critique provided by the reviewer, acknowledging the limitations inherent in our study. This work will serve as an important starting point in addressing the issue of aspiration pneumonia, and we acknowledge that further investigations are essential to build upon our initial findings. The limitations highlighted by the reviewer are duly noted, and we are committed to addressing these shortcomings in future research endeavors.

Moving forward, we are actively planning and working toward conducting more comprehensive studies with larger sample sizes and a multi-center approach. This collaborative effort will undoubtedly enhance the generalizability and impact of our research. We thank the reviewer for their insightful comments, and we are dedicated to refining our methodologies and study design to contribute substantively to the understanding of aspiration pneumonia.
