# Supplementary material for: Effect of one dose of ceftriaxone during endotracheal intubation on the incidence of aspiration pneumonia in cerebral hemorrhage patients: A randomized, controlled, double-blind clinical study protocol
Source: PLoS One. 2025 Jan 14;20(1):e0316811. doi: 10.1371/journal.pone.0316811 (PMC11731750; doi:10.1371/journal.pone.0316811)
Supplement: Attachment — Submitted filename: Response to Reviewers.docx [file pone.0316811.s004.docx]

Dear *PLOS One* Editors,

Thank you for your thoughtful comments and suggestions for our manuscript, “Effect of one dose of ceftriaxone during endotracheal intubation on the incidence of aspiration pneumonia in cerebral hemorrhage patients: A randomized, controlled, double-blind clinical study protocol.” Below in blue are responses to Reviewer comments that you will also find addressed in our manuscript. We appreciate the opportunity to address the comments and resubmit an improved manuscript for considered publication in *PLOS One*.

Sincerely,

Xuan Song, MD, PhD

Reviewer #3: Dear Authors, thank you for giving me the opportunity to review this article.
However, I still have questions about the study.
Would you like to perform a subgroup analysis regarding mortality prognosis for patients with intracranial hemorrhage?

We extend our sincere gratitude to the reviewer for their positive and constructive review of our manuscript. We have included a secondary outcome analysis of 28-day mortality for patients with intracranial hemorrhage (see Research Outcomes).

Would the cost-effectiveness analysis be the same in the geriatric population with comorbid diseases with high mortality rates?

No cost-effectiveness analysis was performed in designing this study. However, a single dose of ceftriaxone will very likely be more cost-effective than aspiration pneumonia treatment in any population.

Again, as a subgroup analysis, should the low-risk patient group be evaluated as the same as the patient group with visible stomach contents during intubation, aspiration suspected, and evidence of aspiration by bronchoscopy?

Yes, we will perform a subgroup analysis of low-risk and high-risk patients based on intubation characteristics (see Research Outcomes). Low risk patients will be defined as those with none of the following: visible stomach contents during intubation, aspiration suspected, and evidence of aspiration by bronchoscopy. High-risk patients will be defined as having one or more of the following: visible stomach contents during intubation, aspiration suspected, and evidence of aspiration by bronchoscopy.

I think that the methodology of the study should be reorganized with a solid justification and a good subgroup analysis.
Kind Regards

Reviewer #4: I thank the editor for giving me the opportunity to review the manuscript entitled Effect of one dose of ceftriaxone during endotracheal intubation on the incidence of aspiration pneumonia in cerebral hemorrhage patients: A randomized, controlled, double-blind clinical study protocol by Xuan Song and others for the second time. The reviewer #5 has made some comments and sought some clarification to which authors have responded. I have gone through the comments and reply given by the authors. Reviewer# 5 pointed in his comments that that the definition of pneumonitis and Pneumonia has to made clear to which the authors have given the explanation which is adequate Present study protocol deals with aspiration pneumonia hence chemical pneumonitis needn't be included secondly the most probable organism responsible for aspiration pneumonia are from throat secretion mostly consists of gram positive and few gram negative organisms sensitive to ceftriaxone. hence the selection of antibiotic is justified thirdly the authors agreed to reviewer comment that multicentric trial should be carried out basing on the result of study to be carried out by present protocol The authors have modified manuscript as per the reviewers comment and I am satisfied with the modification Hence I recommend that the manuscript may be accepted without any further modification

We extend our sincere gratitude to the reviewer for their positive and constructive review of our manuscript. Your insightful comments and feedback have been invaluable, and we appreciate the time and effort you dedicated to evaluating our work.

Reviewer #6: thank you very much for giving me the opportunity to review the manuscript" Effect of one dose of ceftriaxone during endotracheal intubation on the incidence of aspiration pneumonia in cerebral hemorrhage patients: A randomized, controlled, double-blind clinical study protocol". I think the main issue with this work is the antimicrobial resistance (AMR) that may happen with the blind consumption of antibiotics that you have mentioned in your exclusion criteria that if you see any sign of regurgitation you will exclude the patients. it means, you don't have any clue for an aspiration and still you use ceftriaxone as a prophylaxis.

We extend our sincere gratitude to the reviewer for their constructive review of our manuscript. We admit that this study may increase the use of antibiotics in patients who might otherwise have not received them, but only one dose of cephalosporin will be used. For these patients, the benefits of preventing aspiration pneumonia may outweigh the small risk for AMR. To address the concern of AMR, we will compare the intensity of antibiotic use in the secondary outcomes.

We have excluded patients who exhibit regurgitation as these patients may already be exhibiting symptoms of aspiration pneumonia. We plan to study ceftriaxone as a prophylactic measure to prevent aspiration pneumonia before it develops. Thus, we must exclude patients who may have already developed aspiration pneumonia.

Secondly, you have mentioned that you will exclude your patients if they receive any antibiotic during 48 hours after the surgery. the question is this: is it possible for a patient not to receive antibiotic after those surgeries?

The only post-procedure antibiotic patients in this study receive will be ceftriaxone. We have excluded patients who receive other post-procedure antibiotics because the use of additional antibiotics may confound the results.

Thirdly, I even didn't see the result part of this manuscript. you have skipped result and started discussion after method.

This manuscript describes a protocol that has not yet been performed, so there are no results to report at this time.

Reviewer #7: The revised manuscript is interesting and good written and discussed.
Introduction was good written
Materials and methods were good written
Results were good written
Discussion was good written

We extend our sincere gratitude to the reviewer for their positive and constructive review of our manuscript. Your insightful comments and feedback have been invaluable, and we appreciate the time and effort you dedicated to evaluating our work.
