# Supplementary material for: Effect of one dose of ceftriaxone during endotracheal intubation on the incidence of aspiration pneumonia in cerebral hemorrhage patients: A randomized, controlled, double-blind clinical study protocol
Source: PLoS One. 2025 Jan 14;20(1):e0316811. doi: 10.1371/journal.pone.0316811 (PMC11731750; doi:10.1371/journal.pone.0316811)
Supplement: Attachment — Submitted filename: Response to Reviewers.docx [file pone.0316811.s005.docx]

Dear *PLOS One* Editors,

Thank you for your thoughtful comments and suggestions for our manuscript, “Effect of one dose of ceftriaxone during endotracheal intubation on the incidence of aspiration pneumonia in cerebral hemorrhage patients: A randomized, controlled, double-blind clinical study protocol.” Below in blue are responses to Reviewer comments that you will also find addressed in our manuscript. We appreciate the opportunity to address the comments and resubmit an improved manuscript for considered publication in *PLOS One*.

Sincerely,

Xuan Song, MD, PhD

Reviewer #3: Dear authors, I have reviewed your article for re-evaluation.
But I still have some questions that I can't get answers to.
First of all, the dates between April 2023 and April 2024 should be changed.

We have changed the dates to between April 2023 and April 2025.
Could you please state more clearly why patients with intracranial hemorrhage were selected?

Patients with intracerebral hemorrhage often experience complications which require endotracheal intubation to protect the airway and maintain oxygenation. During endotracheal intubation, the application of analgesic sedative drugs and glottis exposure may lead to aspiration and aspiration pneumonia. The primary objective of this study is to explore whether a single dose of ceftriaxone administered during endotracheal intubation in patients with intracerebral hemorrhage reduced the incidence of pneumonia within 48 hours. Our work will provide evidence for the prevention of aspiration pneumonia in patients with intracerebral hemorrhage who undergo endotracheal intubation. This is the first clinical study of ceftriaxone to prevent the development of aspiration pneumonia in patients with intracerebral hemorrhage undergoing endotracheal intubation.
For Introduction:
Please give the necessary message by simplifying the numbers in the second paragraph.

We have simplified the numbers in the second paragraph by limiting the reported numbers to aspiration pneumonia only and reporting only the one-year mortality rate from Yoon *et al.,* 2019.
Likewise, there are too many names in the fourth paragraph. Simplify and combine this paragraph with the next paragraph, that is, your purpose for choosing Ceftriaxone.

We have simplified the fourth paragraph, combined it with the fifth paragraph, and removed the names.
Discussion: In the second paragraph, only information is given.
Information that should be included in the introduction section.

We have moved the information from the second paragraph in the Discussion to the Introduction.
Create a relationship with your work and state what your work will add with this information.
The third and fourth paragraphs likewise contain only information.
Things I hope to see in the discussion section:
Which gap in the literature will be closed with your study?

This is the first clinical study of ceftriaxone to prevent the development of aspiration pneumonia in patients with ICH hemorrhage undergoing endotracheal intubation.
What is the reason for the conflicting information in the literature regarding your choice of ceftriaxone? What is the difference of your study?

Other studies used different antibiotics and more than one dose. We decided to use a single dose of ceftriaxone, an antibiotic which has a long half-life and is effective against the main pathogens responsible for most endotracheal intubation-induced aspiration pneumonia. We hypothesize that a single dose of this drug will reduce the risk of subsequent infection by resistant microorganisms.

Our study will also be randomized, as opposed to other, non-randomized studies that are present in the literature.
Why were ICH patients chosen? What kind of deficiency is there in the literature regarding this?

Patients with intracerebral hemorrhage often require endotracheal intubation to protect the airway, but this procedure can lead to aspiration and subsequent aspiration pneumonia. This study is the first to explore ceftriaxone's role in preventing aspiration pneumonia in intracerebral hemorrhage patients, addressing a gap in the literature.
Your sample size is quite small. Your results will not be strong for mortality.

We have acknowledged our small sample size as a limitation in the Discussion.
Additionally, as a comparison in your study; I recommend that you compare the risk of aspiration pneumonia in intubated patients with ICH in the year before the study in your hospital with the group receiving ceftriaxone in your study.
Please rewrite this beautiful study protocol by enriching the discussion section.
Kind Regards

We have endeavored to improve the discussion section to address the above points. Specifically, we moved much of the information to the Introduction and made sure to clarify what sets our study apart from existing literature.

Reviewer #4: Thanks for giving me the opportunity to review the manuscript which I have already reviewed before, and found it fit for publication. I feel no further modification is required as I am satisfied with the responses given by authors to the reviewers comments and agreeing to incorporate these aspects in the study protocol.

We extend our sincere gratitude to the reviewer for their positive and constructive review of our manuscript. Your insightful comments and feedback have been invaluable, and we appreciate the time and effort you dedicated to evaluating our work.
