# Supplementary material for: Effect of one dose of ceftriaxone during endotracheal intubation on the incidence of aspiration pneumonia in cerebral hemorrhage patients: A randomized, controlled, double-blind clinical study protocol
Source: PLoS One. 2025 Jan 14;20(1):e0316811. doi: 10.1371/journal.pone.0316811 (PMC11731750; doi:10.1371/journal.pone.0316811)
Supplement: Attachment — Submitted filename: Response to Reviewers.docx [file pone.0316811.s006.docx]

Dear *PLOS One* Editors,

Thank you for your thoughtful comments and suggestions for our manuscript, “Effect of one dose of ceftriaxone during endotracheal intubation on the incidence of aspiration pneumonia in cerebral hemorrhage patients: A randomized, controlled, double-blind clinical study protocol.” Below in blue are responses to Reviewer comments that you will also find addressed in our manuscript. We appreciate the opportunity to address the comments and resubmit an improved manuscript for considered publication in *PLOS One*.

Sincerely,

Xuan Song, MD, PhD

Reviewer #8: The sample size needs to be calculated for the protocol. What are the results of the incidence of pneumonia from preliminary data? What test will be used? What attrition rate was assumed?
Thank you for your comments. The sample size calculation will be based on preliminary data, with the expected incidence of pneumonia within 48 hours after intubation, and we will use the two-sample rate comparison formula. We will also account for an attrition rate to ensure sufficient power for the analysis.
The statistical analysis needs to be rewritten. Clarify the primary endpoint will be compared using chi-square test/fisher exact test. Will demographics/covariates be adjusted for the primary analysis?

We have clarified the statistical analysis in the manuscript. The primary endpoint, the incidence of aspiration pneumonia, will be compared using the chi-square test or Fisher’s exact test, and demographics and relevant covariates will be adjusted for in the primary analysis using regression techniques.

Reviewer #9: 1) The statement “This is a prospective, randomized, controlled, double-blind clinical study, which was reviewed by the ethics committee of Dong E Hospital…” should come under study design.

We have moved this statement to the appropriate place in the manuscript.
2) The paragraph that begins with “Patients with ICH undergoing endotracheal intubation in Dong E Hospital of….” Should be captioned inclusion and exclusion criteria

We have added the appropriate heading to this paragraph in the manuscript.
3) Sample size calculation. The authors indicated that a total of 50 patients will be included in the preliminary study. What informed the number 50? How many patients will be in the control, and how many will be in the intervention? Estimation of sample size for clinical trial should be based on the minimum detectable effect (expected change) after the implementation of the intervention, the type of hypothesis, design effect to account for correlation of patient response at the facility level, attrition rate, type I error, power of the study. The sample size of 50 was assumed, and a more detailed sample size must be conducted so that we determine the number of patients required. The section on sample size, which is a critical component of the protocol, is missing. This is also not a pilot study but a full clinical trial protocol.

The sample size of 50 was initially based on preliminary data; however, we will conduct a more detailed sample size calculation for the full clinical trial based on the minimum detectable effect, type of hypothesis, design effect, attrition rate, and statistical power. The final number of patients required for the control and intervention groups will be determined through this calculation.
4) Statistical Analyses of the proposed study must highlight a detailed analytical plan, including how each outcome variable (primary and secondary outcomes) will be measured (continuous, discrete, nominal, ordinal outcomes) and the specific statistical test that will be applied including potential assumption of the test and how violations of the assumptions will be addressed. The current write-up is all lumped together with no clear pattern as which statistical analyses will be used to address the study hypothesis.

We have revised the statistical analysis section to provide a more detailed and structured approach, specifying how each outcome variable (primary and secondary) will be measured (whether continuous, discrete, nominal, or ordinal) and the specific statistical tests that will be applied. Additionally, we have addressed the assumptions of the tests used and outlined how violations of these assumptions will be handled to ensure clarity and rigor in the analysis plan.
